# Sedative–Hypnotic Activity of the Water Extracts of *Coptidis Rhizoma* in Rodents

**DOI:** 10.3390/clockssleep4010014

**Published:** 2022-03-04

**Authors:** Hye-Young Joung, Minsook Ye, Miyoung Lee, Yunki Hong, Minji Kim, Kyung Soo Kim, Insop Shim

**Affiliations:** 1 Department of Physiology, College of Medicine, Kyung Hee University, Seoul 02435, Korea; hyjyj007@hanmail.net (H.-Y.J.); yunkizz@naver.com (Y.H.); 2 Department of Integrative Medicine, College of Medicine, The Catholic University of Korea, Seoul 29612, Korea; jh486ms22@naver.com (M.Y.); kskim@catholic.ac.kr (K.S.K.); 3 KM Convergence Research Division, Korea Institute of Oriental Medicine, 1672 Yuseong-daero, Daegu 41062, Korea; mylee@kiom.re.kr; 4 Department of East West Medical Science, Graduate School of East-West Medical Science, Kyung Hee University, Seoul 02447, Korea; dor9240@nate.com

**Keywords:** *Coptidis Rhizoma*, sedative–hypnotic activity, GABA_A_–BZD receptor, 5-HT_2C_ receptor, arylalkylamine *N*-acetyltransterase (AANAT), pentobarbital-induced sleep test, sleep architecture

## Abstract

Many medicinal plants have been used in Asia for treating a variety of mental diseases, including insomnia and depression. However, their sedative–hypnotic effects and mechanisms have not been clarified yet. Accordingly, the objective of this study was to investigate the sedative–hypnotic effects of water extracts of five medicinal plants: *Coptidis Rhizoma*, *Lycii Fructus*, *Angelicae sinensis Radix*, *Bupleuri Radix*, and *Polygonum multiflorum Thunberg*. The binding abilities of five medicinal plant extracts to the GABA_A_–BZD and 5-HT_2C_ receptors were compared. Their abilities to activate arylalkylamine *N*-acetyltransferase (AANAT), a melatonin synthesis enzyme, in pineal cells were also determined. Following in vitro tests, the sedative and hypnotic activities of extracts with the highest activities were determined in an animal sleep model. In the binding assay, the water extracts of *Coptidis Rhizoma* (WCR) showed high binding affinity to the GABA_A_–BZD and 5-HT_2C_ receptors in a dose-dependent manner. Additionally, WCR increased the AANAT activity up to five times compared with the baseline level. Further animal sleep model experiments showed that WCR potentiated pentobarbital-induced sleep by prolonging the sleep time. It also decreased the sleep onset time in mice. In addition, WCR reduced wake time and increased non-rapid eye movement (NREM) sleep without EEG power density (percentages of δ, θ, and α waves) during NREM sleep in rats. WCR could effectively induce NREM sleep without altering the architectural physiologic profile of sleep. This is the first report of the sedative–hypnotic effect of *Coptidis Rhizoma* possibly by regulating GABA_A_ and 5-HT_2C_ receptors and by activating AANAT activity.

## 1. Introduction

Sleep disorder or sleep loss can cause the dysregulation of homeostasis and lead to the impairment of immune functioning, which could be a significant factor contributing to a variety of inflammatory and chronic diseases. Approximately 15% of the adult population suffers from long-term insomnia, and these values are still higher among adults with concomitant medical or psychiatric disorders [1]. At present, therapy for sleep disorders includes sedative–hypnotic medications, such as benzodiazepines (BZD)/non-BZD (GABA_A_ receptor agonists), sedating antidepressants (tricyclic antidepressant, 5-hydroxytryptamine receptor antagonists), and antihistamines [2]. however, drugs, including hypnotic medications generally used for treating sleep disorders, are associated with side effects, such as mood disorders, lung diseases, and cognitive deficits [3,4,5,6,7,8]. Therefore, interest in herbal drugs as alternatives to prescribed medicine is increasing to enhance the quality of sleep and avoid adverse outcomes.

Commonly available herbal remedies for sleep disorders in the West are as follows: *St John’s wort* (*Hypericum perforatum*), valerian (*Valeriana officinalis*), hops (*Humulus lupulus*), and skullcap (*Scutellaria laterifolia*) [2,9]. Korean traditional medicine has a long history of medical practice based on the cumulative knowledge of herbal plants as sources of therapeutic herbal drugs [8,10]. So far, sedative–hypnotic efficacies of medicinal plants, such as *Schisandra sphenanthera* and *Euphoria longana*, have been reported in Asia [11,12]. Several medicinal plants have also been used to improve sleep disturbance in Korea. However, research studies about their sedative–hypnotic effects are insufficient. *Coptidis Rhizoma*, *Lycii Fructus*, *Angelicae sinensis Radix*, *Bupleuri Radix*, and *Polygonum multiflorum Thunberg* have long been used as oriental medicine in the treatment of a variety of psychiatric disorders, including anxiety and insomnia, although studies reporting their efficacies are lacking. The molecular target of medicinal plants has been mainly focused on GABAergic and serotonergic regulation in the CNS, which is known to be involved in various mental functions related to relaxation, stress, and sleep regulation [12,13]. GABA was reported to strike a neuronal balance between excitation and inhibition [14]. Several findings have implicated the GABAergic system in the control of sleep, and the BZD binding site on the GABA_A_ receptor was considered as one of the most crucial targets for sedative–hypnotic drugs development [13,14,15]. BZDs can stimulate the opening of Cl^−^ influx using GABA, thus inhibiting neurotransmission. These agents have common pharmacological properties, such as sedative–hypnotic, anxiolytic, and anticonvulsant effects [13,15]. Additionally, it was reported that *Coptidis Rhizoma* and its main component, namely, berberine, have antidepressant-like activities via serotonergic neurotransmission.

The molecular targets of medical plants for sedative activity have been mainly focused on the serotonergic system, which is known to possess various mental functions related to relaxation, stress, and sleep regulation. The modulation of serotonin at the synapse is thought to be a major action of several classes of pharmacological antidepressants. In addition, 5-HT_2A_, 5-HT_2B_, and 5-HT_2C_ receptors were demonstrated to play an important role in various CNS actions, such as mood, sleep, and satiety [16]. Particularly, the 5-HT_2C_ receptor was reported to be a potential target for sedative–hypnotic or anxiolytic drugs [17]. Previous studies reported that the GABA_A_ receptor is modulated by the 5-HT_2C_ receptor-mediated elevation of Ca^2+^ [18,19].

Melatonin (*N*-acetyl-5-methoxytryptamine) is synthesized enzymatically de novo in pineal glands from serotonin by arylalkylamine *N*-acetyltransterase (AANAT) and hydroxy-indole-O-methyltransferase [20,21]. AANAT is involved in the first step of the pathway that synthesizes melatonin from serotonin [22]. Melatonin was reported as a potential treatment for immune diseases, depressive disorder, circadian rhythm sleep disorder, and insomnia in older adults [23,24,25]. Despite the known essential role of AANAT activation in melatonin synthesis, sedative–hypnotic medicinal plant studies using AANAT assays are insufficient. Thus, we investigated the effects of herbal candidates on the serotonin/melatonin and GABAergic systems and their possible mechanisms. The purpose of this research was to verify five medicinal plants (*Coptidis Rhizoma*, *Lycii Fructus*, *Angelicae sinensis Radix*, *Bupleuri Radix*, and *Polygonum multiflorum Thunberg*) as herbal sedative–hypnotic candidates through GABA_A_–BZD- and 5-HT_2c_-receptor-binding assays and AANAT assays using pineal cells. Their sleep-promoting effects were then confirmed using animal sleep models.

## 2. Results

### 2.1. Binding Affinity of Medicinal Plants Extracts to the GABA_A_–BZD Receptors

**Figure 1:**
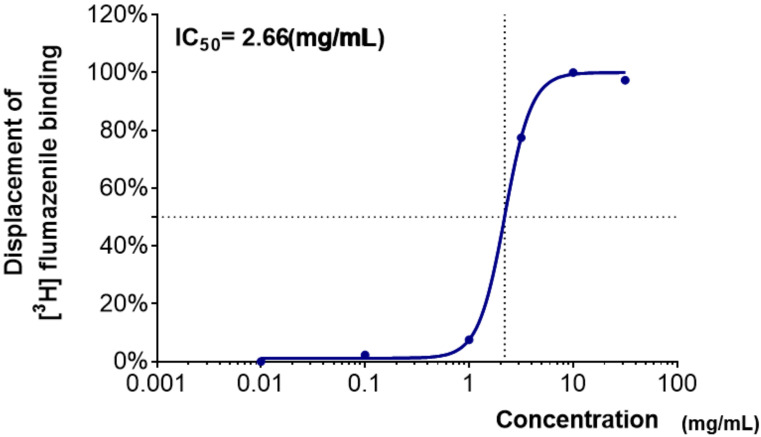
Dose–response curve of WCR in the GABA_A_–BZD-receptor-binding assay. Each data point represents the mean ± SD (*n* = 3). Abbreviation: WCR, water extract of *Coptidis Rhizoma*.

As shown in Table 1, the displacement of [^3^H]-flumazenil (GABA_A_–BZD antagonist) binding was obtained for water extracts of medicinal plants at six concentrations. WCR showed the highest binding affinity to the GABA_A_–BZD receptors, which replaced over 80% of the [^3^H]-flumazenil binding at a concentration of 20 mg/mL. The IC_50_ value of WCR was 2.66 mg/mL (Figure 1). The water extract of *Polygonum multiflorum* *Thunberg* (WPmT) had a weaker but dose-dependent binding activity to GABA_A_–BZD receptors. Moreover, water extracts of *Bupleuri Radix* (WBR) showed medium dose-dependent binding affinity to GABA_A_–BZD receptors.

### 2.2. Binding Affinity of Plant Extracts to the 5-HT_2C_ Receptors

**Figure 2:**
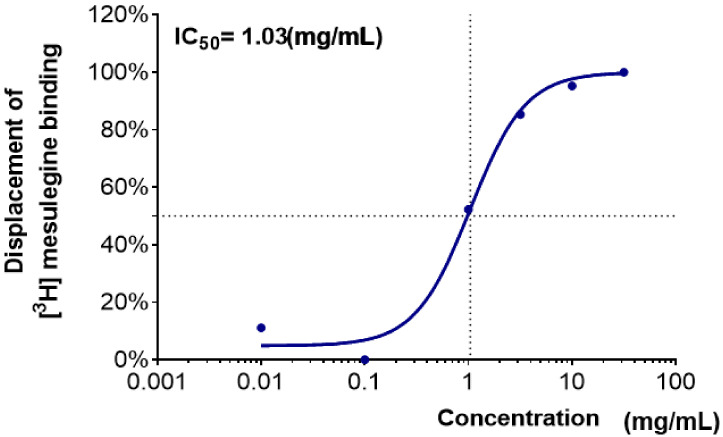
Dose–response curve and half-maximal inhibitory concentration (IC_50_, mg/mL) values of WCR in the 5-HT_2C_-receptor-binding assay. Each data point represents the mean ± SD (*n* = 3). Abbreviation: WCR, water extract of *Coptidis Rhizoma*.

The binding activities of water extracts to the 5-HT_2C_ receptor are shown as the percentage displacement of [^3^H]-mesulergine (5-HT_2C_ agonist) binding (Table 2). As in the GABA_A_–BZD-receptor-binding assay, water extracts of *Lycii Fructus* (WLF) and *Angelicae sinensis Radix* (WAsR) did not show an effective binding affinity. WBR, which showed an effective binding affinity to the GABA_A_–BZD receptor, did not show any effective binding activity to the 5-HT_2C_ receptor. WPmT had weak binding to the 5-HT_2C_ receptor at 10 mg/mL. On the other hand, the WCR showed effective binding activity. The IC_50_ value of WCR was 1.03 mg/mL (Figure 2).

### 2.3. AANAT Assay

**Figure 3:**
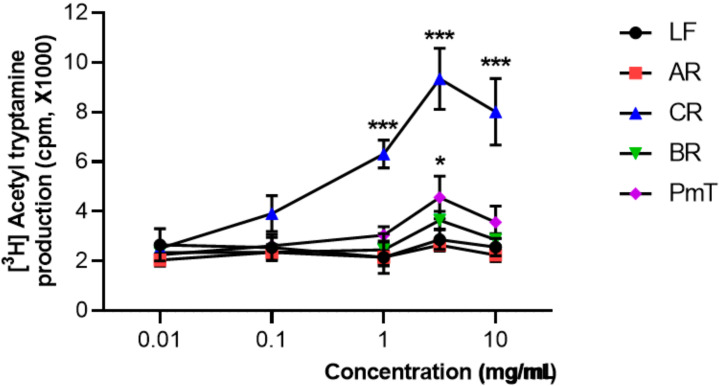
The effect of water extracts of medicinal plants on AANAT activity in the pineal cells. Each data point represents the mean ± SD (*n* = 3). Abbreviations: LF, *Lycii Fructus*; AR, *Angelicae sinensis Radix*; WCR, water extracts of *Coptidis Rhizoma*; BR, *Bupleuri Radix*; PmT, *Polygonum multiflorum* *Thunberg*. Statistical analysis was performed using one-way ANOVA followed by Tukey’s test. * *p* < 0.05, *** *p* < 0.001, significantly different compared with the baseline level.

The effects of water extracts of medicinal plants on the AANAT activity in the pineal cells are shown in Figure 3. The activity of AANAT was expressed in terms of the control activity. Treated WCR (*n* = 3) robustly and dose-dependently increased the AANAT enzyme activity in pineal cells compared with the baseline level (0.1~100 mg/mL, *** *p* < 0.001, significantly different compared with the baseline level, respectively).

### 2.4. The Effect of WCR on Pentobarbital-Induced Sleep in Mice

**Figure 4:**
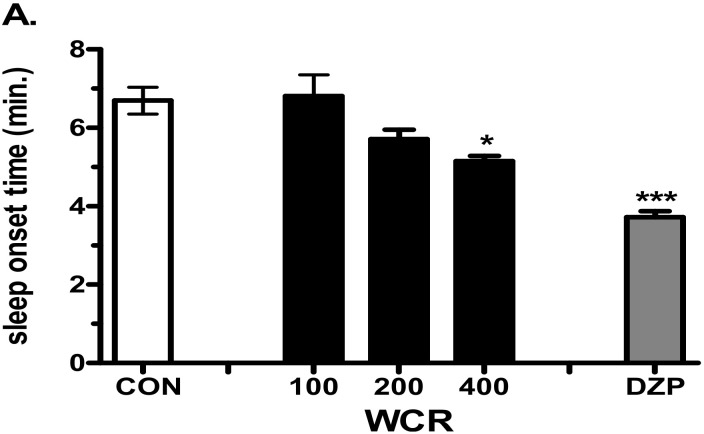

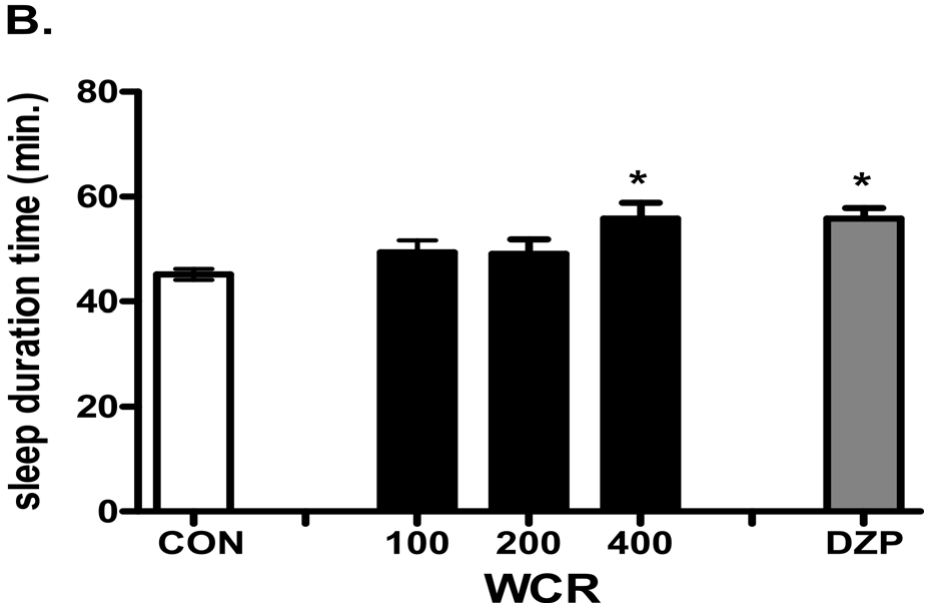
Effects of WCR on the onset time of sleep (**A**) and sleep duration time (**B**) in mice induced with sedative–hypnotic pentobarbital (30 mg/kg). Each column displays the mean ± SEM. Statistical analysis was conducted using one-way ANOVA followed by Tukey’s test. * *p* < 0.05, *** *p* < 0.001, significantly different compared with the CON group. Mice were injected with pentobarbital 30 min after treatment with WCR or DZP (Diazepam). The CON group: treatment with vehicle and pentobarbital sodium (30 mg/kg); the WCR groups: treatment with WCR (100 or 200 or 400 mg/kg) and pentobarbital sodium (30 mg/kg); the DZP group: treatment with diazepam 2 mg/kg and pentobarbital sodium (30 mg/kg).

Effect of the injection of WCR in a pentobarbital-induced sleep mouse model is presented in Figure 4. The experimental group was split into the following subgroups: control (CON, *n* = 10), WCR 100 mg/kg (100, *n* = 10), WCR 200 mg/kg (200, *n* = 10), WCR 400 mg/kg (400, *n* = 10), and DZP 2 mg/kg treatment (DZP, *n* = 10) in the pentobarbital test. ANOVA indicated a significant effect of WCR on the sleep onset time (F(4,49) = 15.80, *p* < 0.001) and the sleep duration time (F(4,49) = 5.657, *p* < 0.01). As expected, DZP (2 mg/kg) significantly potentiated the pentobarbital-induced sleep in mice. WCR generated a dose-dependent decreasing tendency in sleep latency and increasing tendency of sleep duration at the sedative–hypnotic dose of pentobarbital (30 mg/kg). Lower doses (100 and 200 mg/kg) of WCR had no significant sedative–hypnotic effect. However, a high dose (400 mg/kg) of WCR had a significant (*p* < 0.05) sedative–hypnotic effect.

### 2.5. Effect of WCR on the EEG Sleep Architecture in Rats

**Figure 5:**
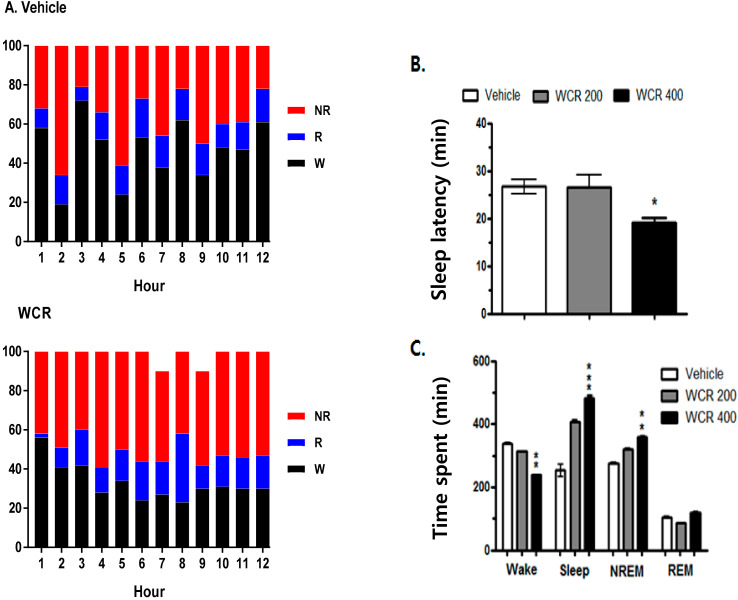
(**A**) Sleep–wake pattern in rats treated with the vehicle or WCR. (**B**) Effect of WCR on sleep latency. (**C**) Time spent in wake, total sleep, NREM sleep, and REM sleep after WCR administration. Each column represents the mean ± SEM. * *p* < 0.05, ** *p* < 0.01, *** *p* < 0.001, significantly different compared with the vehicle group. ANOVA followed by Tukey’s test. The CON group: treatment with the vehicle; the WCR groups: treatment with WCR (200 or 400 mg/kg).

The effect of WCR on the EEG sleep architecture and profile are displayed in Figure 5. The experimental group was split into the following subgroups: control (CON, *n* = 5), WCR 100 mg/kg (100, *n* = 5), WCR 200 mg/kg (200, *n* =5), WCR 400 mg/kg (400, *n* = 5), and DZP 2 mg/kg treatment (DZP, *n* = 5) regarding the EEG sleep architecture. ANOVA indicated a significant effect of WCR on the sleep latency time (F(3,17) = 5.337, *p* < 0.05), time spent in wake (F(3,17) = 16.8, *p* < 0.001), total sleep (F(3,17) = 6.48, *p* < 0.001), NREM sleep (F(3,17) = 4.12, *p* < 0.001), and REM sleep (F(3,17) = 2.089, *p* = 0.1583). The EEG and EMG signals in rats were registered for 12 h post WCR treatment (200 and 400 mg/kg, p.o.) at 08:00 AM. WCR significantly reduced the sleep latency (Figure 5B). No change in REM sleep was observed in all the tested rats during the 12 h recording time (Figure 5C). WCR (400 mg/kg) induced a significant reduction in the sleep onset time and an increase in the sleep duration time.

**Figure 6:**
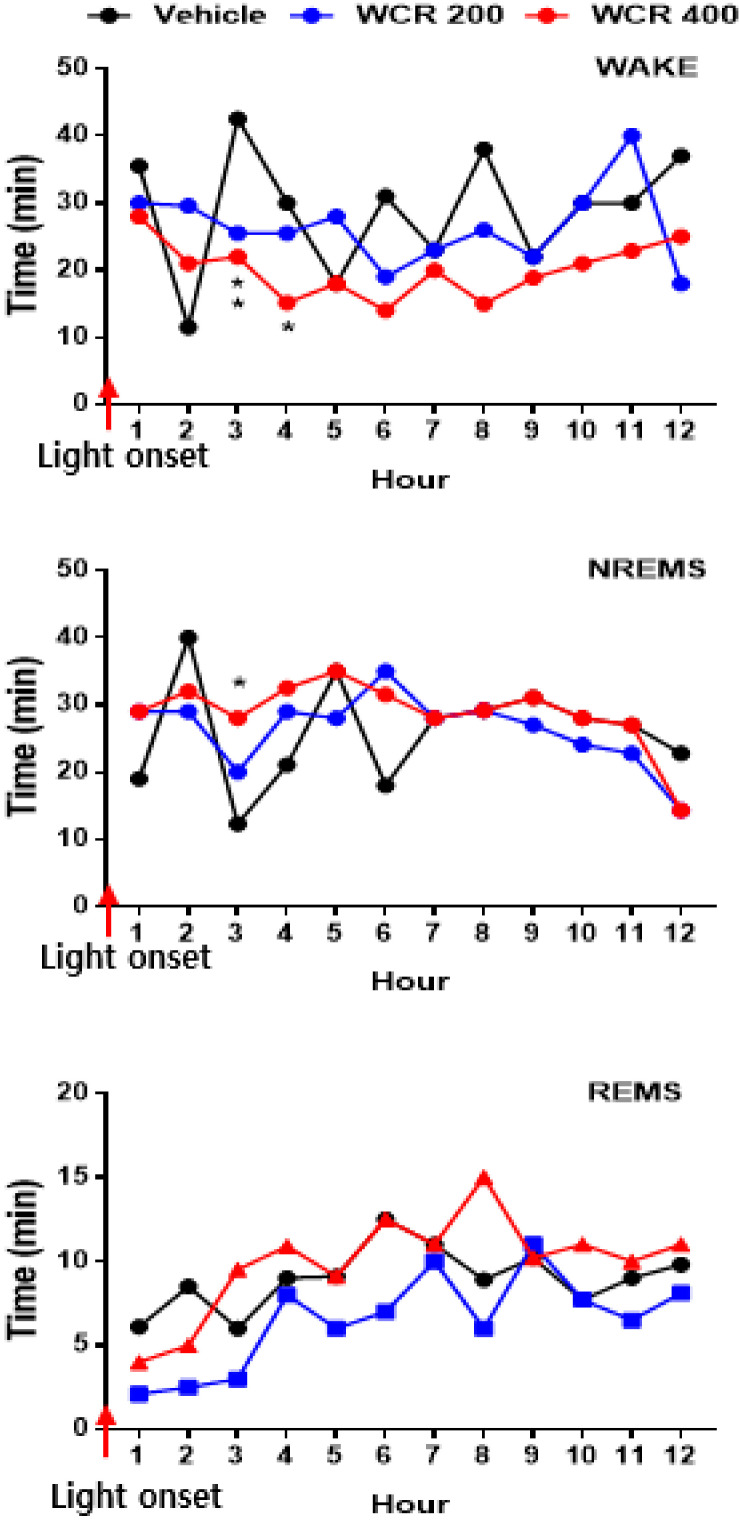
Time course of wake, NREM sleep, and REM sleep after treatment with WCR. Each circle displays the hourly mean ± SEM. * *p* < 0.05, ** *p* < 0.01, significantly different compared with the vehicle group. ANOVA followed by Tukey’s test. The CON group: treatment with the vehicle; the WCR groups: treatment with WCR (200 or 400 mg/kg).

The time courses of the hourly amounts of wake, NREM sleep, and REM sleep for 12 h post WCR treatment are presented in Figure 6 (F(3,17) = 7.8, *p* < 0.01). A significant decrease in the hourly amount of wake (*p* < 0.05) and a significant increase in the hourly amount of NREM sleep (*p* < 0.01) were observed for the first 3 h after the WCR treatment. However, there was no significant change in REM sleep after the WCR treatment.

**Figure 7:**
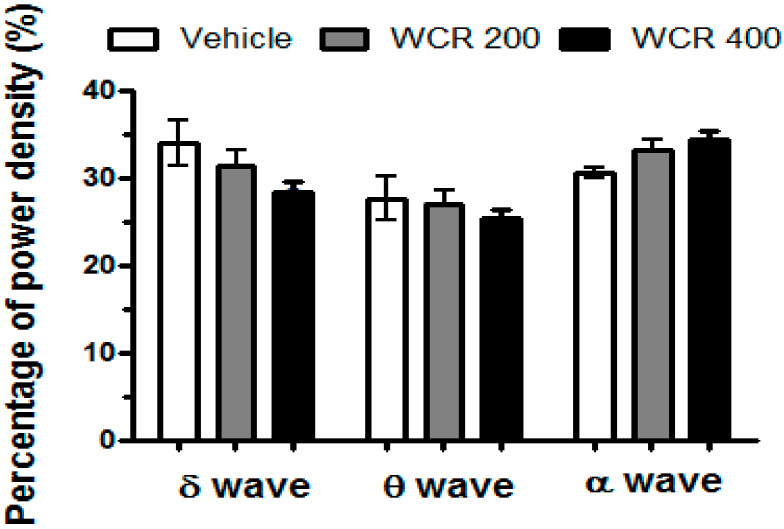
Effects of WCR on the EEG power during NREM sleep. The EEG power densities during delta, theta, and alpha wave activities for NREM sleep were measured. The data displays the mean ± SEM of the EEG power density in three selected frequency bands in the NREM sleep stage. The CON group: treatment with the vehicle; the WCR groups: treatment with WCR (200 or 400 mg/kg).

The effects of WCR on the EEG power density during NREM sleep are presented in Figure 7 (F(2,12) = 0.1944, *p* = 0.9808). During NREM sleep, WCR did not induce any changes in the EEG power density of all three selected frequency bands.

## 3. Discussion

In the present study, five medicinal plants with expected sedative–hypnotic activity were chosen to evaluate the sedative–hypnotic effects of their water extracts. Their binding activities to 5-HT_2C_ and GABA_A_–BZD receptors and their effects on AANAT activity in pineal cells were determined. Using water extracts of medicinal plants with high binding activity and enhanced AANAT activity, an animal sleep model was adopted to confirm their sedative–hypnotic effects. WCR showed binding activity to GABA_A_–BZD and 5-HT_2C_ receptor and AANAT activity. It also enhanced pentobarbital-induced sleep in the animal model. These results suggest that the hypnotic activity of WCR might be due to the binding of 5-HT_2C_ and GABA_A_ receptors and AANAT activity.

A receptor-binding assay using a radioligand is very useful for drug discovery. GABA_A_–BZD- and 5-HT_2C_-receptor-binding assays have been largely used to screen for centrally acting drugs with sedative–hypnotic and anxiolytic activities [26,27]. In the present study, the most active candidate among the five medicinal plants was WCR. WCR showed a moderate dose-dependent binding affinity to the GABA_A_–BZD receptor (IC_50_ value of 2.66 mg/mL) and 5-HT_2C_ receptor (IC_50_ value of 1.03 mg/mL). These results indicated that WCR contained at least two natural ligands that could bind differently to GABA_A_–BZD and 5-HT_2C_ receptors. WCR’s binding affinity appears to occur in multiple ways due to various sedative–hypnotic activities exerted by its constituent bioactive compounds. However, the other water extracts had a weak binding activity only at the highest dose. This could be due to the low activity of the extract or non-specific inhibitory interaction with other components of plant extracts.

The possible mechanism of WCR on sleep latency may have been through modulating the central serotonergic and GABAergic systems. In the present study, WCR showed high binding affinity to 5-HT_2C_ receptors in a dose-dependent manner. This result suggests that WCR increased 5-HT levels in the sleep-promoting regions, such as the pineal gland and hypothalamus. This suggestion is supported by the fact that the injection of berberine, which is one of the WCR components, can increase the levels of serotonin in mouse brains [28]. Accumulating evidence and the present results indicated that an increase in 5-HT may promote sleep through the activation of 5-HT receptors. It was shown that pinealocytes contain 5-HT and its receptors, which is one of the most important neurotransmitters for its role in regulating sleep–wake homeostasis [22]. Our data demonstrated the first experimental evidence that WCR has sedative efficacy, suggesting WCR as a novel natural medication to enhance sleep by modulating serotonergic systems and activating sleep-promoting regions in the brain. *Coptidis Rhizoma* (*Coptis chinensis Franch*; CR) as an ingredient of Whangryunhae-dok-Tang, which contains a combination of *Phellodendri Cortex*, *Scutellariae Radix*, and *Gardeniae Fructus*, has been prescribed by Oriental medicine doctors in Korea for treating mental disorders patients with insomnia. The chemical constituents of CR are mainly alkaloid (berberine), protoberberine alkaloids (coptisine, worenine, palmatine), polysaccharide, and organic acids. Many clinical and experimental studies reported its various biological properties, such as anticancer, antioxidant, anti-inflamatory, and neuroprotective effects, as well as neurological inhibitory effects on the CNS [29,30]. Berberine, the main component of WCR, is known to produce a variety of biological effects on the central nervous system. It was reported that CR and berberine can produce antidepressant-like activities via serotonergic neurotransmission [31,32]. Injection of berberine can increase levels of serotonin in mouse brains [28] and also decrease immobility time and reverse depressive-like effects in ovariectomized mice. This effect is blocked by the 5-HT_2_ antagonist ketanserin [33]. Therefore, the sedative efficacy of WCR may be due to the actions of berberine.

Because AANAT is known to control the night/day rhythm of melatonin production in the pineal gland, which plays critical roles in the circadian regulation of sleep–wake cycles, as it attenuates wake-promoting signals of the suprachiasmatic nucleus (*SCN*) while promoting the consolidation of sleep, we investigated the effects of WCR on AANAT activity. AANAT catalyzes the rate-limiting step in the biosynthesis of melatonin from serotonin in the pineal gland. Stimulated AANAT activity is also potentially therapeutic for various mental diseases, such as sleep and mood disorders [34,35]. It regulates seasonal reproduction in mammals, reptiles, and songbirds, and it controls migration behavior. The hormone melatonin has been implicated in sleep and circadian clock function. Large changes in the abundance of AANAT are believed to be responsible for the day/night rhythms of melatonin synthesis in the pineal gland and retina. Most related studies showed that AANAT activity is regulated by controlling steady-state levels of the protein. Our results showed that WCR dose-dependently enhanced AANAT enzyme activity in a robust manner, suggesting that WCR could increase melatonin synthesis. The pineal gland is well known as a photo-neuro-endocrine organ situated inside the brain, releasing serotonin and melatonin [25,26,27]. The pineal gland secretes the hormone melatonin from pinealocytes throughout the night. The secretion has a circadian rhythm, primarily because it is driven by the nocturnal release of norepinephrine (NE) from pinealopetal sympathetic nerve fibers of the superior cervical ganglia [36,37]. NE acting through α_1_- and β_1_-adrenergic receptors activates the expression of the rate-limiting enzyme of AANAT, in turn converting 5-HT into *N*-acetylserotonin (NAS), and a second enzyme hydroxyindole O-methyltransferase generates melatonin [38]. Circadian regulation of melatonin is known to involve multi-synaptic connections that extend from the SCN to the pineal gland. During the dark phase of the cycle, the paraventricular nucleus (PVN) projects to the preganglionic cells in the intermediolateral cell column of the spinal cord, which promotes the release of NE from cells in the superior cervical ganglia that project to the pineal gland. Activation of β_1_-adrenergic receptors in the pinealocytes results in protein kinase A activation and, subsequently, an increase in the enzymatic activity of AANAT. We could not exclude the possibility that WCR activates the β_1_ adrenergic receptors on pinealocytes, triggering complex intracellular molecular cascades that eventually regulate melatonin synthesis. Further study is needed to examine the effects of WCR on β_1_-adrenergic receptors.

In the present study, WCR also showed a high binding affinity to the GABA_A_–BZD receptor in a dose-dependent manner. GABA release is important in maintaining inhibitory tone, which also plays an important role in sleep and anxiety. GABA_A_ receptors, which are a family of ligand-gated chloride ion channels, mediate the effect of GABA in sleep-related behavior. Previous studies reported that the GABAergic system is involved in the hypnotic action of melatonin. For example, melatonin is known to increase the concentration of GABA in the hypothalamus [39], potentiate GABA turnover, and cause an enhancement of GABA binding [40].

Taken together, these results suggest that WCR produces its hypnotic and anxiolytic-like effects through interaction with the GABA_A_ and 5-HT_2c_ receptors, both of which are known to play a critical role in sleep functions. This suggestion is consistent with other reports showing that the co-administration of *Glycyrrhiza uralensis Fischer* has a high affinity for the GABA-A receptor, with 5-HT_2C_ receptor producing a strong hypnotic effect, and is supported by a previous study that showed that the 5-HT_2C_ receptor is co-localized with GABAergic neurons. In addition, it was shown that GABAergic neurons are regulated by the activation of 5-HT_2C_ receptors [15].

It is important for active ingredients from medicinal plants to cross the blood–brain barrier (BBB) for a sedative–hypnotic role. Several lines of evidence suggested that novel antiepileptic and anticonvulsant herbal compounds with a binding activity on the GABA_A_ receptor could pass the BBB [15,41]. Although the results of the binding assay indicated that WCR might contain natural ligands for GABA_A_ and 5-HT_2C_ receptors, we did not determine the possibility of BBB penetration. Additionally, previous studies reported that the systemic administration of berberine, one of the main compounds of CR, could cross the BBB and show multiple therapeutic actions in CNS disorders [42]. Thus, we planned to confirm the sedative–hypnotic effect of WCR using animal sleep models. In particular, the pentobarbital-induced sleep test is known to be a useful tool for evaluating inhibitory effects in the CNS, particularly in GABAergic systems [13,18,43]. The results showed that WCR (400 mg/kg) not only prolonged the sleep time induced by pentobarbital (30 mg/kg) but also decreased the sleep onset time in mice. However, the architectural profile and sleep quality could not be assessed using the pentobarbital-induced sleep test. To better determine the sedative–hypnotic activity of WCR, its effects on wake–sleep modulation and profile were investigated via EEG and EMG recordings using rats [11,43]. Some studies showed that the homeostatic demand for the total amount and frequency of sleep depends on specific activity patterns of the cortical EEG wave [44,45,46]. We recorded profiles of sleep–wake using EEG in freely moving rats after WCR administration. WCR increased the amounts of NREM sleep in a dose-dependent manner without significantly changing REM sleep, which is consistent with the belief that WCR would be effective at improving sleep. Change in delta wave activity (or slow-wave activity: spectral power in the 0.75–4.0 Hz range) during NREM sleep has been considered as a physiological criterion of changes in sleep quality and homeostatic sleep intensity [47]. Our results showed that WCR (400 mg/kg) increased NREM sleep without affecting the EEG power density (percentages of δ, θ, and α waves) in rats. These results demonstrated that WCR can induce sleep without altering the architectural physiologic profile of sleep.

We found that treatment with WCR produced a dose-dependent decrease in sleep latency and increase in sleep duration. WCR binds to both GABA_A_–BZD and 5-HT_2C_ receptors and synergistically stimulates 5-HT and GABAergic systems in the brain. These mixed serotonergic and GABAergic mechanisms may be highly involved in modulating the sedative or anxiolytic-like effects of sleeping-promoting WCR. These results suggest that WCR produces its hypnotic and anxiolytic-like effects through the interaction of the GABA_A_ with 5-HT_2c_ receptors, both of which are known to play a critical role in sleep functions.

## 4. Materials and Methods

### 4.1. Materials Extracts

Table 3 presents the scientific names, traditional usage, and plant parts used for extraction. A voucher specimen (DHU-2013-03) of the plant was deposited at the Research Center for Biomedical Research Resources of Medicine, Daegu-Haany University, Dae-gu, Korea. All medicinal plants were purchased from the Daewon oriental medicine (Daegu, Korea). Cut and dried plant materials (3 kg) were powdered and extracted with distilled water for 12 h at 100 °C according to the extraction method of the provider. The resultant extract was condensed using a rotary evaporator (EYELA, Tokyo, Japan) and then lyophilized. The lyophilized powder was dissolved in 0.9% NaCl and stored as stock until use in further experimentation.

### 4.2. Animals

Adult male Sprague Dawley rats (250–300 g) were used to perform a membrane preparation for the GABA_A_–BZD-receptor-binding assay and AANAT assay from the pineal cells. In the pentobarbital-induced sleep test, ICR mice (male 20–25 g) mice were purchased from Orient Bio Co. (Gyeonggi-do, Korea). Room temperature was maintained at 20–25 °C with cycles of 12 h of light and 12 h dark (LD12:12). Food and water were available ad libitum. All rodents were kept for at least 7 days for acclimation. All animal care and testing conditions were in accordance with the Institutional Animal Care and Use Committee (IACUC) in the College of Medicine, the Catholic University of Korea.

### 4.3. [^3^H]-Flumazenil Binding Assay

The GABA_A_–benzodiazepine assay was conducted according to a published study [37] with minor modifications. Briefly, cerebral cortices of rat were homogenized in 20 volumes of Tris-citrate buffer (30 mM, pH 7.4). The suspension was centrifuged at 0–4 °C for 15 min at 27,000× *g*. The pellet was washed three times with 20 volumes of Tris-citrate buffer. After centrifuging at 27,000× *g* for another 15 min, the pellet was resuspended in Tris-HCl buffer (50 mM, pH 7.4, 20 volumes) and incubated in a water bath at 37 °C for 30 min to eliminate the endogenous GABA. The suspension was centrifuged at 27,000× *g* for another 10 min at 0–4 °C. The final pellet was resuspended in 30 mL of Tris-HCl buffer and kept in aliquots at −20 °C until the binding assay. The membrane preparation was thawed and resuspended in Tris-citrate buffer and centrifuged at 27,000× *g* for another 10 min. Washes and centrifugation of the membrane were repeated twice. The pellet was resuspended in Tris-citrate buffer (g of tissue/500 mL binding buffer) and utilized for the binding assay. A test solution (10 µL) was added into the membrane suspension (180 µL) (20–0.01 mg/mL). It was then added with 10 µL of 1 nM of [^3^H]-flumazenil (Ro 15-1788; Perkin Elmer Life and Analytical Sciences Waltham, MA, USA). The mixture was added into a 96-well microplate, blended, and incubated at 0–4 °C for 40 min. To terminate the binding reaction, the mixture was filtered with Whatman GF/C glass fiber filter with ice-cold Tris-HCl buffer (50 mM). These filters were dried at 60 °C for 30 min and suspended in a Wallac 1450 Microbeta plate prior to the addition of scintillation fluid. The radioactivity on the filter was estimated using a liquid scintillation counter in a Wallac 1450 Microbeta plate (Perkin Elmer Life and Analytical Sciences, Waltham, MA, USA). Specific binding was calculated by subtracting the measured non-specific binding (NSB) from the measured total binding (TB). TB and NSB were measured in the presence of 1 mM binding buffer and clonazepam, respectively. The radioligand binding displacement percent was calculated with the following equation.

Data were analyzed using a nonlinear regression program for fitting one-site competitive binding curves (GraphPad Software Inc., San Diego, CA, USA). The half-maximal inhibitory concentration (IC_50_) was then calculated (see Equation (1)):

Binding displacement (%) = [1 − (DPM _sample_ − DPM_NSB_)/DPM_TB_ − DPM _NSB_] × 100 (1)

DPM: disintegrations per minute, NSD: non-specific binding, TB: total binding

### 4.4. [^3^H]-Mesulergine Binding Assay

The 5-HT_2C_-receptor-binding assay was conducted using a human 5-HT_2C_ receptor membrane (Perkin Elmer Life and Analytical Sciences, Waltham, MA, USA) and a 4 µg/mL incubation buffer consisting of 50 mM Tris-HCl (pH 7), 4 mM CaCl_2_, and 0.1% ascorbic acid. Membrane suspension (180 µL) was added to a test solution (10 µL, 0.001–10 mg/mL) and 10 µL (1 nM, final concentration) of [^3^H]-mesulergine (Perkin Elmer Life and Analytical Sciences, Waltham, MA, USA) in a 96-well microtiter plate and blended. The plate was then incubated at 27 °C for 60 min. For the termination of the binding reaction, the mixture was filtered with a Whatman GF/C glass fiber filter with ice-cold Tris-HCl buffer (50 mM). The filter was dehydrated at 60 °C for 30 min and suspended in Wallac Microbeta plate scintillation fluid. The amount of filter-bound radioactivity was counted with a Wallac 1450 Microbeta Liquid scintillation counter (Perkin Elmer Life and Analytical Sciences, Waltham, MA, USA). The specific binding was calculated as TB minus NSB. TB and NSB were determined using a binding buffer and mainserin (100 µM, final concentration), respectively. The percentage displacement of the radioligand binding and IC_50_ values were counted as described above.

### 4.5. Pineal Gland Dissociation Culture

Pineal gland dissociation culture used the method described by Bernard et al. [48] with minor modifications for the preparation of the pineal cell culture. Pineal cells were scattered in trypsin-EDTA and plated in Dulbecco’s modified Eagle’s medium (DMEM, GIBCO, Grand Island, NY, USA) supplemented with 25 mM Hepes buffer, l-glutamine, penicillin, streptomycin, and 10% heat-inactivated horse serum at 37 °C in a humidified atmosphere of 10% CO_2_/90% air. Each six-well plate had cells in groups of four wells. Pineal cells were plated at a density of 1 × 10^6^ cells/well. After 24 h of subculture, the cells were switched to serum-free DMEM for treatment.

### 4.6. AANAT Assay

The AANAT assay was performed as described by Chae et al. [49] with minor modifications. Briefly, cells were collected and washed three times with PBS after treatment with a medicinal plant extract to measure the AANAT activity in a pineal cell culture. After centrifugation, cell lysis was carried out via ultrasound in 80 µL ice-cold phosphate buffer (50 mM, pH 6.8). The remaining solution was eliminated via centrifugation (15,000× *g*, 5 min at 4 °C). The clear supernatant was transferred to a fresh tube and stored at −80 °C until use. An amount of 8 µL of the supernatant was then incubated in the presence of 5 µL tryptamine-HCl (10 mM), 1 µL acetyl-CoA (0.5 mM), 1 µL [^3^H]-acetyl-CoA (3.6 C_i_/mmol, 250 µC_i_/mL), and 10 µL of a test solution (0.001–10 mg/mL). The reactions were incubated at 37 °C in a water bath for 30 min. The reactions were then stopped by diluting the reaction mixture with phosphate buffer (50 mM, pH 6.8). The diluted reaction mixture was rapidly transferred to a vial containing 3 mL of Econofluor (1,2,4-trimethylbenzene (>99 %), 2,5-diphenyloxazole (0.7%, *w*/*v*), or indicated home-made water-immiscible scintillation fluid. The amount of radiolabeled acetyltryptamine was determined in a liquid scintillation counter.

### 4.7. Measurement of Pentobarbital-Induced Anesthesia

The hypnotic assessment method was based on the prolongation of anesthesia induced using pentobarbital [50]. Pentobarbital sodium (Hanlim Pharm. Co., Ltd., Seoul, Korea) was diluted in saline and administered to each mouse (i.p., 30 mg/kg) to induce sleep. The *Coptidis Rhizoma* extract (100, 200, and 400 mg/kg) was suspended in saline and injected orally (p.o.) to animals (10 mL/kg). Animals were not given food for 24 h before the experiment. Thirty minutes after the oral injection of the test sample, pentobarbital was given to animals placed in a box. Those animals that stopped moving in the box within 15 min after the pentobarbital injection were immediately transferred to another box. Individuals that stayed immobile for more than three minutes were judged to be asleep. The sleep latency was recorded from the pentobarbital injection to the sleep onset. Sleeping time was defined as the difference in time between the loss and recovery of the righting reflex.

### 4.8. EEG Surgery

Electroencephalogram (EEG) and electromyogram (EMG) electrodes were implanted for polygraphic recording, as described in the stereotaxic atlas of Paxino and Watson [51]. Rats were anesthetized with pentobarbital (40 mg/kg, i.p.) and chronically transplanted with a head mount. The body of the transmitter was implanted subcutaneously off the midline and posterior to the scapula attached to the skin and sutured three times for stabilization. Electrodes were anchored into the skull with screws and dental cement. All surgical procedures were conducted under aseptic conditions through stereotaxic procedures. After surgery, each rat was allowed 7 days in an individual transparent barrel for recovery.

### 4.9. Methodology of EEG and EMG Recording

After recovery, rats were habituated to the recording cage before the test for at least one week. After oral administration, rats were immediately connected to EEG and EMG recording cables (two EEG channels and one EMG channel). Recording was started at 09:00 a.m. (light/dark 08:00 a.m./08:00 p.m.) and continued for 12 h. Cortical EEG signals were amplified (×100), filtered (low-pass filter, 100 Hz EEG), digitized at a sampling rate of 200 Hz, and recorded with a PAL-8200 data acquisition system (Pinnacle Technology Inc., Lawrence, KS, USA) using a chart speed of 25 mm/s.

Sleep–wake states were automatically categorized into wakefulness (wake), rapid eye movement (REM) sleep, and non-REM (NREM) sleep with SleepSign v. 3 software (Kissei Comtec, Nagano, Japan). Latency of sleep was defined as the time that elapsed between the sample injection and the first consecutive NREM sleep episode lasting at least 2 min and uninterrupted by more than six 4 s epochs not scored as NREM sleep.

### 4.10. Statistical Analysis

For multiple comparisons, behavioral data were analyzed using one-way analysis of variance (ANOVA). Receptor affinity and AANAT data are expressed as means ± SD. Behavioral data are expressed as means ± S.E.M. Statistical differences between groups were further analyzed using Tukey’s post hoc test. The level of significance was set at *p* < 0.05.

## 5. Conclusions

In conclusion, WCR displayed differential binding affinities for GABA_A_ and 5-HT_2C_ receptors and stimulated AANAT activity in pineal cells. WCR also produced sedative–hypnotic activity in the pentobarbital-induced sleep test using mice. Moreover, WCR reduced sleep latency and advanced the amount of NREM sleep without altering REM sleep or the EEG power density during NREM sleep in rats. Taken together, these results suggest that WCR is a good candidate as a herbal agent for treating disturbed sleep.

## Figures and Tables

**Table 1:** Displacement of the [^3^H]-flumazenil binding of water extracts of medicinal plants to the GABA_A_–BZD receptor in the rat cerebral cortex.

Species	Displacement (%) of [^3^H]-Flumazenil Binding at Different Concentrations (mg/mL)					
Scientific Name	0.01	0.1	1	5	10	20
*Lycii Fructus*	−2.1 ± 0.7	−2.1 ± 4.7	−14.6 ± 1.4	−8.6 ± 0.6	−1.5 ± 3.7	10.5 ± 4.6
*Angelicae sinensis Radix*	3.2 ± 1.7	7.5 ± 2.7	−0.8 ± 4.2	5.28 ± 2.3	4.3 ± 1.2	14.5 ± 1.5
*Coptidis Rhizoma*	−6.5 ± 6.2	−4.4 ± 1.2	0.4 ± 1.6	64.2 ± 1.0	84.8 ± 0.6	82.4 ± 0.8
*Bupleuri Radix*	25.0 ± 1.5	26.0 ± 3.8	31.5 ± 2.1	55.3 ± 0.9	62.8 ± 0.5	78.2 ± 0.6
*Polygonum multiflorum* *Thunberg*	−16.8 ± 0.5	−18.9 ± 0.8	−17.9 ± 1.5	−10.0 ± 1.5	0.1 ± 2.5	22.2 ± 1.9

**Table 2:** Displacement of the [^3^H]-mesulergine binding of water extracts of medicinal plants to the membrane expressing the human 5-HT_2C_ receptor.

Species	Displacement (%) of [^3^H]-Mesulergine Binding at Different Plant Concentrations (mg/mL)					
Scientific Name	0.01	0.1	1	5	10	20
*Lycii Fructus*	22.4 ± 0.2	15.2 ± 3.1	13.6 ± 5.4	12.2 ± 4.0	9.3 ± 4.1	13.4 ± 0.8
*Angelicae sinensis Radix*	15.9 ± 4.5	17.8 ± 3.1	8.6 ± 5.4	−2.0 ± 3.0	5.0 ± 11.3	2.2 ± 12.1
*Coptidis Rhizoma*	31.3 ± 3.3	22.8 ± 0.3	62.7 ± 0.2	87.9 ± 1.4	95.5 ± 0.4	99.1 ± 0.2
*Bupleuri Radix*	15.5 ± 4.7	12.8 ± 1.5	18.7 ± 4.7	19.6 ± 0.4	0.4 ± 3.1	−5.7 ± 1.8
*Polygonum multiflorum* *Thunberg*	16.3 ± 2.0	15.6 ± 2.6	14.2 ± 0.9	36.8 ± 1.3	59.2 ± 2.3	66.1 ± 0.4

**Table 3:** Medicinal plant extracts screened for sedative–hypnotic activity.

Species		Plant Part Analyzed	Traditional Usage for Neuropsychology
Name	Scientific Name		
Gouqi	*Lycii Fructus*	Fruit	Inhibition of CNS, sedation
Danghui	*Angelicae sinensis Radix*	Root	Relaxation, sedation
Barberry root	*Coptidis Rhizoma*	Root	Relaxation, treatment of anxiety symptoms
Chai Hu	*Bupleuri Radix*	Root	Sedation, analgesic activity
Huang bai	*Polygonum multiflorum* *Thunberg*	Bark	Anti-inflammation

## Data Availability

Not applicable.
